# Eculizumab in the treatment of Shiga toxin haemolytic uraemic syndrome

**DOI:** 10.1007/s00467-018-4025-0

**Published:** 2018-07-30

**Authors:** Patrick R. Walsh, Sally Johnson

**Affiliations:** 1 Institute of Cellular Medicine, Newcastle University, Newcastle upon Tyne, UK; 2 National Renal Complement Therapeutic Centre, Newcastle upon Tyne, UK; 3 Great North Children’s Hospital, Royal Victoria Infirmary, Sir James Spence Institute, Newcastle, UK; 4 Department of Paediatric Nephrology, Great North Children’s Hospital, Newcastle Upon Tyne Hospitals NHS Foundation Trust, Newcastle upon Tyne, UK

**Keywords:** Haemolytic uraemic syndrome, Eculizumab, Complement, Shiga toxin, STEC-HUS

## Abstract

Haemolytic uraemic syndrome (HUS) remains a leading cause of paediatric acute kidney injury (AKI). Haemolytic uraemic syndrome is characterised by the triad of microangiopathic haemolytic anaemia, thrombocytopenia and AKI. In ~ 90% of cases, HUS is a consequence of infection with Shiga toxin-producing *E. coli* (STEC), most commonly serotype O157:H7. Acute mortality from STEC-HUS is now less than 5%; however, there is significant long-term renal morbidity in one third of survivors. Currently, no specific treatment exists for STEC-HUS. There is growing interest in the role of complement in the pathogenesis of STEC-HUS due to the discovery of inherited and acquired dysregulation of the alternative complement system in the closely related disorder, atypical HUS (aHUS). The treatment of aHUS has been revolutionised by the introduction of the anti-C5 monoclonal antibody, eculizumab. However, the role of complement and anti-complement therapy in STEC-HUS remains unclear. Herein, we review the current evidence of the role of complement in STEC-HUS focusing on the use of eculizumab in this disease.

## Haemolytic uraemic syndrome

Shiga toxin-producing *E. coli* (STEC) infection, most commonly serotype O157:H7 [1], results in haemorrhagic colitis in the majority of children infected. In 85–90% of cases, this resolves with no further sequelae within 1 week of onset. In 10–15% of infected children, haemolytic uraemic syndrome (HUS) develops, most commonly 2 weeks after the onset of the colitis [2]. The hallmark features of HUS are a triad of microangiopathic haemolytic anaemia, thrombocytopenia and acute kidney injury (AKI) [3]. This clinical presentation occurs due to acute thrombotic microangiopathy (TMA), most commonly in the renal microvasculature. The characteristic histological features in the renal microvasculature include mesangiolysis, endothelial swelling and fibrin-rich thrombi (often with fragmented erythrocytes) within the glomeruli [4]. In addition to the renal involvement, extra-renal manifestations occur in approximately 20% of cases [5]; the most devastating of these is neurological involvement. While not as common as renal involvement, neurological dysfunction represents the major cause of mortality in HUS [6–8]. The majority of children with HUS recover with best supportive care; this includes temporary dialysis in approximately 50–75% and red cell transfusion in 80% [9–12].

## Pathophysiology

**Fig. 1:**
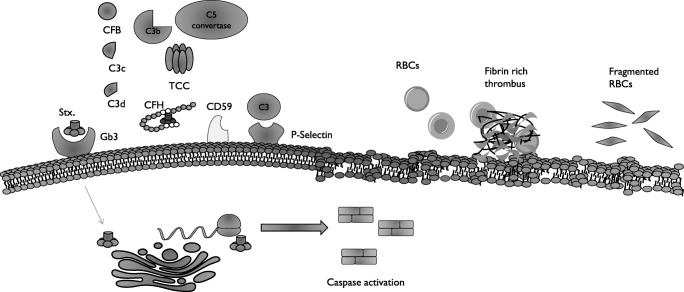
Proposed mechanism of Shiga toxin resulting in thrombotic microangiopathy (TMA). On binding to the Gb3 receptor, *stx*. is internalised and trafficked through the Golgi apparatus where it is released in to the cytoplasm; *stx*. then binds to the ribosome and blocks transcription resulting in activation of apoptotic pathways. Ultimately, this results in activation of platelet and the endothelium. As this process continues thrombi fill and occlude the capillary lumen resulting in mechanical haemolysis as erythrocytes are forced through these fibrin-rich thrombi. The role of complement activation in this process is unclear. Observational data from patients with Shiga toxin-producing *E.coli* (STEC-HUS) demonstrates increased plasma levels of the complement component C3b, factor B and the C5 convertase (C3bBbC3b) as well as C3 breakdown products C3c and C3d. Further to this, *stx.* is able to bind to complement factor H (CFH), which leads to impaired complement regulation on the cell surface. Evidence of terminal complement pathway dysregulation is evidenced by the increased circulating membrane attack complex and decreased CD59 mRNA, a regulator of the membrane attack complex. *Stx.* has been shown to upregulate surface expression of P-selectin; this receptor is able to capture circulating C3 and promotes thrombus formation

Shiga toxin-producing *E.coli*  are highly infectious organisms, with an estimated infective dose as low as ten organisms [13], compared to > 10^5^ organisms required for infection from other *E. coli* species [14]. Most commonly, STEC infection occurs as a result of ingestion of contaminated food or water [15]. Shiga toxin-producing* E.coli* possesses a number of properties that increase its virulence; firstly, intrinsic acid resistance enables survival through the acidic environment of the stomach [16]. Once through the stomach, STEC must colonise the intestinal mucosa; this is achieved through a number of specialised proteins encoded on the locus of enterocyte effacement and ultimately result in attaching and effacing (A/E) lesions [17]. These lesions result in loss of microvilli and accumulation of actin within the host cell, anchoring the bacteria to the surface. Once adhered to the intestinal mucosa, STEC begin producing Shiga toxin (*stx.*) [18]. Shiga toxin is a member of the ribosome inactivating protein (RIP) family, made up of one A chain, responsible for inactivating ribosomal activity, and five identical B chains that aid binding to the target receptor [19]. Shiga toxin-producing *E.coli* is capable of producing two *stx.* (*stx1* and *stx2*); while structurally similar, individuals infected with *stx2* are more likely to develop HUS [20]. Once secreted, *stx.* transverses the intestinal wall and enters the bloodstream, a process which is not yet fully understood [21]. Within the bloodstream, *stx.* binds to circulating polymorphonuclear leukocytes and is transported to distal sites [22]. The main cellular target for *stx.* is the globotriaosylceramide (Gb3) receptor located on the microvascular endothelium within the brain, gut and kidney [21]. Within the kidney, in addition to the endothelium, Gb3 is expressed on the surface of tubular cells, mesangial cells and, in primates, podocytes [23]. Once bound to Gb3, *stx.* enters the cell via endocytosis and is trafficked through the Golgi apparatus and endoplasmic reticulum, before being released into the cytosol [18]. Once in the cytosol, *stx.* exerts its effect via inhibition of the ribosomal activity and subsequent blockage of protein transcription. These events lead to activation of apoptotic pathways, induction of inflammatory cytokines and cellular necrosis [21] (Fig. 1). All these processes lead to the generation of a pro-inflammatory environment within the microvasculature. The role of the complement system in this process is discussed hereafter.

## The complement cascade

The complement system is a complex cascade of over 30 proteins that together forms part of the innate immune system [24]. It is composed of three pathways, namely the classical, alternative and lectin-binding. The alternative pathway is constitutively active at a low level via spontaneous hydrolysis of circulating C3 molecules generating C3(H_2_O) (a process known as C3 tick-over) [25]. This hydrolysis results in a conformational change in C3, which permits its interaction with factor D, the resulting complex cleaves factor B (CFB) to Ba and Bb. C3(H_2_O) and Bb complex to generate the C3 convertase (C3bBb), which binds to the cell surface and forms the basis of the C3 amplification loop, whereby the C3 convertase is able to cleave further C3 to C3a (anaphylatoxin) and C3b. C3b generated by this process binds to the C3 convertase, forming the C5 convertase (C3BbC3b) [26]. The C5 convertase cleaves circulating C5 to C5a (anaphylatoxin) and C5b. Finally, C5b complexes with C6, C7, C8 and C9 forming the membrane attack complex (MAC). This structure forms a permeable pore in the cell membrane leading to massive fluid and electrolyte shift resulting in cell lysis. To prevent over-activity of the pathway and to protect host cells from damage by complement, a number of fluid phase (Complement Factors H (CFH), I (CFI)) and membrane-bound (CD46, DAF and CD59) regulators exist.

## The role of complement in STEC-HUS

Complement activation was first observed in STEC-HUS over 30 years ago, when it was demonstrated that children with STEC-HUS had higher plasma levels of the alternative complement activation products, C3b, C3c, C3d and factor B [27, 28]. These findings have been reproduced and extended more recently with evidence of increased levels of C5 convertase and the common endpoint of complement activation soluble C5b-C9 (or terminal complement complex, TCC), a fluid phase form of MAC [29–31]. Further evidence of complement involvement in STEC-HUS is supported by the presence of circulating complement-containing microvesicles from platelets, leukocytes and erythrocytes in individuals with STEC-HUS [32, 33], suggesting a direct interaction between these cells and complement. Together, these observations indicate the alternative complement cascade is activated during STEC-HUS. The centrality of *stx.* in the pathogenesis of STEC-HUS is evident, but the extent to which the observed alternate complement pathway activation contributes to the morbidity and mortality of this disease remains unclear.

## Possible mechanisms of complement activation in STEC-HUS

Perhaps the most direct evidence of complement activation by *stx2* has been provided by Orth et al. [34]. In this study, incubation of *stx.* with normal human serum resulted in increased TCC, indicating activation of the final common complement pathway. This increased activation was seen with co-incubation with EGTA (a classical pathway blocker), but not with EDTA (which blocks complement activation completely), signifying *stx2* activates complement via the alternative pathway. In the same study, *stx2* was shown to bind CFH. Complement factor H is composed of 20 homologous short consensus repeat (SCR) units; these units are highly conserved containing approximately 60 amino acids that are arranged in a ‘bead on string’ orientation. *stx2.* binds specifically to SCR 6–8 and 18–20, the regions responsible for host surface recognition; binding to these regions was shown to reduce surface complement regulation, while fluid phase regulation was preserved [34]. In a separate study, levels of CD59, a regulator of the MAC, were shown to be lower in glomerular endothelial cells treated with *stx2* but not in tubular cells, due to reduced mRNA [35]. These results indicate that *stx2* may result in increased susceptibility to complement-mediated damage in patients with STEC-HUS through a reduction in complement regulation by CFH and CD59.

In addition to the loss of complement regulation, there is evidence of increased microvascular endothelial complement activation after incubation with *stx2.* Following incubation with *stx2*, there is significant upregulation of P-selectin on the surface of an immortalised endothelial cell line, human microvascular endothelial cells (HMEC-1)*.* P-selectin is able to bind and activate C3, leading to increased thrombus formation [36]. Supporting the role of P-selectin in thrombus formation is the finding that an anti-P-selectin antibody reduced both the C3 deposition and thrombus burden in this HMEC-1 model. Interestingly, the use of a C3a receptor (C3aR) antagonist also reduced the thrombus formation and podocyte loss [36, 37], suggesting a role for C3a in the pathogenesis of STEC-HUS.

Taken together, the evidence from both clinical and laboratory studies points to complement activation during STEC-HUS pathogenesis.

## Eculizumab

Eculizumab is a humanised monoclonal IgG2/4 that binds C5, preventing its conversion to C5a and C5b [38]. Prevention of this step effectively blocks the formation of the terminal complement pathway and the MAC. Eculizumab was first approved in 2007 for use in paroxysmal nocturnal haemoglobinuria and subsequently in 2011 for aHUS. Available safety data suggests that eculizumab is effective and safe for the treatment of aHUS in both adults and children [39–41] although there is a significantly increased risk of meningococcal disease due to terminal complement blockade.

## Eculizumab in STEC-HUS

There is no controlled data investigating the use of eculizumab in STEC-HUS; consequently, the only available data on its efficacy comes from small cases series and uncontrolled observational data. In 2011, the first reported use of eculizumab in STEC-HUS was published [42] in which three young children with STEC-HUS, complicated by dialysis-dependent AKI and severe neurological involvement, were treated with eculizumab after deterioration on conventional treatment. All three patients showed dramatic improvement in their neurological symptoms within 24 h of the first dose and normalisation of their haematological parameters within 5 days. These outcomes are impressive, but it is possible the recovery in these patients was unrelated to the use of eculizumab. This report was published just prior to a large outbreak of STEC O104 in Germany [43]. On the basis of this report, Deutsche Gesellschaft für Nephrologie (German Society of Nephrology) recommended eculizumab for the most severely affected patients; this included patients with AKI stage III [44] (serum creatinine three times baseline or greater than 353 μmol/l and/or urine output < 0.3 ml/kg/h or anuria for 12 h), neurological symptoms or thromboembolic events [45]. In a rapidly convened, industry-sponsored, open-label non-randomised trial, 193/491 (39%) adult patients were treated with eculizumab after failure to respond to plasma exchange [46]. The outcomes for some of these patients have been reported [46, 47]. No benefit of eculizumab over best supportive care or plasma exchange was found even after adjustment for potential confounding factors. A parallel observational study was conducted in children affected by O104 HUS [48]. In contrast to the adult study, only 13/90 (14%) children were treated with eculizumab, seven after plasma exchange and six as first line management. Data for sub-groups (eculizumab versus plasma exchange and eculizumab) were not reported separately due to the small number of children treated with eculizumab. However, short-term and intermediate analysis of both this and a separate German cohort has demonstrated no benefit of eculizumab compared to standard care in previous outbreaks [49]. It is difficult to draw any concrete conclusions from these studies. Firstly, in the adult study, patients treated with supportive care alone were significantly older (54.5 vs. 44 years old) and less likely to have neurological symptoms (45% vs. 89%) than the group treated with eculizumab. Secondly, there was a delay of 11 days between presentation and commencing eculizumab treatment; available data from patients with aHUS suggests that early initiation of eculizumab is associated with a better prognosis [41]. It is possible that this delay resulted in reduced efficacy of eculizumab; in fact, it has previously been demonstrated that evidence of complement activation returns to baseline, in patients with STEC-HUS, after approximately 1 week [31]. All patients in the adult study were treated with plasma exchange prior to eculizumab. Plasma exchange has previously been used in patients with STEC-HUS with the rationale of removing circulating bacterial toxin, inflammatory mediators and pro-thrombotic factors. This practice may also have been influenced by the efficacy of plasma exchange in some patients with aHUS. While there is no data supporting any benefit of plasma exchange in STEC-HUS [50], it may be that plasma exchange in these patients reduced the effectiveness of eculizumab, for example by removing complement regulators. One concern about the use of eculizumab is an increased risk of bacterial infection due to loss of the terminal complement pathway [51]. While this risk appears to be most significant for Neisserial infection, there is a theoretical risk that blocking the terminal complement pathway in active STEC infection may increase the risk of invasive sepsis from *E. coli* still bound to the intestinal wall. In the absence of a controlled comparison of eculizumab in STEC-HUS, it is difficult to draw any certain conclusions about the safety or efficacy of eculizumab in STEC-HUS.

During this German outbreak, a group of nine patients in France contracted O104:H4 HUS [52]. All nine were treated with eculizumab, the three index patients received plasma exchange as first line, followed by eculizumab, while the remaining six were treated with eculizumab on presentation. The data presented suggests the group treated with eculizumab at presentation had a milder degree of AKI, lower peak lactate dehydrogenase (LDH) and less thrombocytopenia compared to the patients initially treated with plasmapheresis. It is difficult to judge whether these effects are due to the early initiation of eculizumab or simply that these patients had milder disease. Analysis of the data reveals that while the platelet count improved within 48 h and normalised by 1 week of eculizumab; both the haemoglobin and creatinine were slower to recover. The median haemoglobin continued to fall for 1 week post-eculizumab, while more patients had AKI stage III 7 days after eculizumab. This clinical trajectory could be considered the natural history and variability of the condition. The follow-up data available from this study, 10 weeks post-eculizumab, demonstrates impaired renal function (eGFR 60–90 ml/min/1.73m^2^) in 3/9 patients, new hypertension in 2/9 and albuminuria in one patient. Again, these results are comparable to those seen in patients treated with best supportive care in other cohorts [53].

With the lack of available controlled data on the role of eculizumab in STEC-HUS, there have been a number of case reports and small case series reporting the use of eculizumab in STEC-HUS with neurological involvement [54–56]. Despite the potentially devastating consequences of neurological involvement in STEC-HUS, there is scant evidence regarding the short- and long-term prognosis following this complication. The largest cohort describing the outcomes of children with severe neurological involvement reported 52 children and demonstrated a 17% acute mortality rate. At follow-up, 23% had severe residual impairment; only 50% recovered fully [57]. With these poor outcomes and lack of proven treatment, investigators have been keen to study the potential of anti-complement therapy in these cases. Gitiaux et al. [54] reported seven children with STEC-HUS complicated by neurological involvement who were treated with eculizumab. Brain magnetic resonance imaging (MRI) performed at presentation demonstrated reversible changes. At 6 months, these changes had resolved and neurological testing was normal in the surviving patients (5/7); however, renal function remained impaired in 3/5 children. Pape et al. [56] described 11 children with confirmed STEC and neurological symptoms (seizures 11/11 and stupor/coma 10/11) who received eculizumab. All children required dialysis and one child died from multi-organ failure. Magnetic resonance imaging during the acute presentation was abnormal in 8/10 patients. Dialysis was continued for a median of 15.5 days (4–23) and normalisation of platelet count occurred at 4 days (0–20) after eculizumab. At discharge, one child had severe neurological impairment and three demonstrated mild impairment. At 6 months, 9/10 patients had normal neurological examination, with the remaining child showing substantial improvement. No renal outcomes were reported in these children. These two reports show resolution of neurological symptoms after eculizumab and offer a potentially promising treatment for children with of neurological involvement in STEC-HUS. In the absence of controlled studies, it is not possible to determine whether this effect is due to eculizumab or the highly variable natural history of STEC-HUS.

## Conclusion

A specific treatment for STEC-HUS has remained elusive, despite many attempts [50]. The discovery that complement blockade effectively controls TMA in aHUS, coupled with evidence that complement activation during STEC-HUS, has led to the ad hoc use of eculizumab in adults and children with STEC-HUS, with no systematic assessment of its efficacy or safety. In the absence of randomised controlled trials, it is impossible to conclude if there is truly a beneficial effect from eculizumab in STEC-HUS. Two double-blinded placebo controlled trials (Table 1) [ECULISHU in France looking at renal outcome (NCT02205541) and ECUSTEC in the UK looking at overall disease severity (ISRCTN89553116)] seek to provide evidence to guide use of this therapy in STEC-HUS.

**Table 1:** Summary of two randomised controlled trials investigating the use of eculizumab in STEC-HUS: ECULISHU based in France and ECUSTEC based in the UK

	ECULISHU	ECUSTEC
Patient population	1 month–18 years	6 months–18 years
Diagnosis of HUS	• Mechanical haemolytic anaemia (haemoglobin < 10 g/dl, haptoglobin below reference range, LDH and/or bilirubin above reference range, presence of schistocytes) • Thrombocytopenia (platelets < 150 × 10^9^/l) • AKI defined by an estimated creatinine clearance < 75 ml/min/1.73 m^2^ [58]	• Microangiopathic haemolytic anaemia (indicated by fragmented red cells on blood film *or* LDH above reference range) • Thrombocytopenia (platelets < 150 × 10^9^/l) • AKI: at least ‘injury’ category of pRIFLE criteria [59] despite correction of hypovolaemia
Identification of Shiga toxin *E. coli*	Prodromal diarrhoea and/or presence of an enterohaemorrhagic strain of *E. coli* and/or identification of the *Stx* 1 or 2 genes in the stool sample or rectal swab	Diarrhoea within 14 days prior to diagnosis of HUS or a stool culture/Shiga toxin PCR result indicating STEC in the patient or household contact within 14 days prior to diagnosis of HUS
Exclusion criteria:	• Patient affected by aHUS or family history of aHUS • Neonatal HUS • Malignancy • Pregnancy or lactation • Identified drug exposure-related HUS • Infection-related HUS • Patient with ongoing meningococcal infection • Known HIV infection • Known systemic lupus erythematosus or antiphospholipid antibody positivity or syndrome	• Family history of aHUS • Previous episode of HUS • Pre-existing eGFR < 90 ml/min/1.73m^2^ • Pregnancy • Patient taking a drug known to be associated with HUS, e.g. calcineurin inhibitors, chemotherapy, quinine, oral contraceptive pill • Known or suspected pneumococcal infection
Extra-renal involvement at diagnosis	Exclusion if any of the following symptoms are present at diagnosis: • Neurological involvement (seizures, coma, focal deficit) with signs of microangiopathy on cerebral MRI • Cardiac involvement (cardiac failure, ischemic myocarditis, conduction or rhythm troubles) • Digestive involvement (severe pancreatitis defined by lipasemia > 500 IU/l, severe hepatitis defined by transaminase > × 10 ULN and/or prothrombin time < 60%, haemorrhagic colitis, bowel perforation, rectal prolapse)	No exclusion based on extra-renal involvement
Intervention	Eculizumab or placebo	Eculizumab or placebo
Dosing schedule	3 to 5 injections at D0, D7, D14, D21 and D28	2 injections at D1 and D8
Primary outcome	Duration of renal replacement therapy	Purpose-developed, multi-domain clinical severity score. A single score is assigned at day 60 to reflect cumulative morbidity up until that point
Secondary outcome	• Death • Duration of the thrombocytopenia • Duration of the haemolytic anaemia • Adverse events • Adverse reactions related to the treatment • Duration of AKI • Renal sequelae including blood pressure, creatinine clearance, ionogram, proteinuria and microalbuminuria • Parameters of complement activation: C3 and CD46 • Inhibition of TCC • Neurological involvement (seizures, coma, focal deficit) • Cardiac involvement (cardiac failure, ischemic myocarditis, conduction or rhythm troubles) • Digestive involvement (pancreatitis, hepatitis, haemorrhagic colitis, bowel perforation, rectal prolapse)	• Death • Duration of thrombocytopenia • Duration of haemolysis • Duration of dialysis (days) • CKD at 1 year (a composite endpoint of the presence of hypertension, albuminuria or eGFR < 90 ml/min/1.73 m^2^) • Number of packed red blood cell transfusions required and volume (ml/kg) • Markers of inflammation (number of days until normal total white cell count and CRP) • Persistent neurological defect at 1 year • Health-related quality of life

*LDH* lactate dehydrogenase; *AKI* acute kidney injury; *aHUS* atypical haemolytic uraemic syndrome; *pRIFLE* paediatric risk, injury, failure, loss, end stage renal disease criteria; *Stx* Shiga toxin; *HUS* haemolytic uraemic syndrome; *PCR* polymerase chain reaction; *STEC* Shiga toxin producing *E. coli*; *HIV* human immunodeficiency virus; *eGFR* estimated glomerular filtration rate; *MRI* magnetic resonance imagining; *ULN* upper limit of normal; *TCC* terminal complement complex; *CKD* chronic kidney disease; *CRP* C reactive protein
